# Function and clinical application of exosome—how to improve tumor immunotherapy?

**DOI:** 10.3389/fcell.2023.1228624

**Published:** 2023-08-21

**Authors:** Siwen Qin, Jilong Cao, Xiaoxue Ma

**Affiliations:** ^1^ Department of Pediatrics, The Fourth Hospital of China Medical University, Shenyang, China; ^2^ Party Affairs and Administration Office, The Fourth Hospital of China Medical University, Shenyang, China; ^3^ Department of Pediatrics, The First Hospital of China Medical University, Shenyang, China

**Keywords:** tumor, immunotherapy, exosome contents, exosome membrane molecules, engineered exosomes

## Abstract

In recent years, immunotherapy has been increasingly used in clinical practice to treat tumors. However, immunotherapy’s efficacy varies between tumor types and patient populations, and long-term drug resistance often occurs during treatment. Therefore, it is essential to explore the molecular mechanisms of immunotherapy to improve its efficacy. In this review, we focus on the significance of tumor-derived exosomes in the clinical treatment of tumors and how modifying these exosomes may enhance immune effectiveness. Specifically, we discuss exosome components, such as RNA, lipids, and proteins, and the role of membrane molecules on exosome surfaces. Additionally, we highlight the importance of engineered exosomes for tumor immunotherapy. Our goal is to propose new strategies to improve the efficacy of tumor immunotherapy.

## 1 Introduction

Despite significant medical and technological advances, cancer remains one of the most challenging diseases. The prognosis of patients with cancer is primarily determined by metastasis and recurrence, which often occur after initial treatment (Ganesh and Massague, 2021). According to data from the Global Cancer Observatory (GCO), the number of cancer-related deaths is estimated to reach 30 million by 2030 (The, 2018). In addition to the high mortality rate associated with cancer, the economic burden that cancer patients and their families bear is also substantial.

Moreover, cancer imposes a significant economic burden on societies (The, 2018; Mao et al., 2022). Therefore, efforts to prevent, diagnose, and treat cancer are paramount. The combination of radiotherapy and chemotherapy remains the primary treatment modality for tumors. However, chemoradiotherapy can result in serious side effects and drug resistance, which may be associated with the presence of cancer stem cells (CSCs) (Dagogo-Jack and Shaw, 2018; Pilie et al., 2019). Developing immune checkpoint inhibitors, such as PD-L1 or PD-1 monoclonal antibodies, has improved treatment strategies for certain tumors. These therapies have demonstrated greater efficacy than traditional chemoradiotherapy with fewer side effects (Iwai et al., 2017; Ai et al., 2020; Gou et al., 2020; Jiang et al., 2021). Despite the significant clinical efficacy of immunotherapy in many types of cancer, the response rate in patients remains lower than 40%, and the underlying mechanisms are still not fully understood (Topalian et al., 2015; Jensen et al., 2021). As a result, improving immune efficacy has become an area of focus for further exploration.

Recently, exosomes have emerged as a promising avenue for immunotherapy. Extracellular vesicles (EVs), including ectosomes and exosomes, are released by all types of cells in nature, both prokaryotes and eukaryotes, as part of their normal physiology (Zhou Y. et al., 2021). The outward budding of the plasma membrane forms ectosomes and includes microvesicles, microparticles, and more giant vesicles ranging from approximately 50 nm to 1 μm in diameter (Seo et al., 2018; Wang et al., 2019). Exosomes are a significant class of EV, typically ranging in size from 40 to 200 nm in diameter. They can package various constituents, including mRNA, proteins, non-coding RNA, and metabolites (Morrissey and Yan, 2020; Zhou et al., 2020).

Exosomes interact with recipient cells through receptor-mediated endocytosis or by fusing directly with the plasma membrane of recipient cells and releasing their contents (Kalluri and LeBleu, 2020). Exosomes can induce tumor immune escape through various mechanisms. For instance, it has been found that extracellular vesicles derived from tumor cells carry immunosuppressive molecules such as transforming growth factor-beta (TGF-β), interleukin-10 (IL-10), and indoleamine 2,3-dioxygenase (IDO) (Zhang and Yu, 2019). These factors inhibit the activation and function of immune cells, leading to immune suppression and tumor immune evasion. Additionally, exosomes can influence the phenotype and function of immune cells in the tumor microenvironment, thereby altering antigen presentation, impairing activation and proliferation of T cells, inducing regulatory T cell differentiation, and promoting expansion of myeloid-derived suppressor cells, which creates an immune-suppressive environment that favors tumor growth and escape (Paskeh et al., 2022). For example, exosomes derived from dendritic cells express major histocompatibility complex II (MHC-II), which can be internalized by activated T cells via receptor-mediated endocytosis, thereby modulating the immune efficiency of immunotherapy (Nolte-'t Hoen et al., 2009). Ultimately, exosomes can impact the expression and function of immune checkpoint molecules such as programmed cell death ligand 1 (PD-L1) on both tumor cells and immune cells (Shen and Zhao, 2018; Korman et al., 2022). This modulation can suppress anti-tumor immune responses and promote immune evasion.

This article provides a comprehensive overview of tumor-derived extracellular exosomes’ role in clinical cancer therapy and explores various strategies to modify exosomes to enhance their immunotherapeutic effects. These strategies include changing the content of exosomes, such as RNA, lipids, and proteins, and identifying membrane molecules on exosomes. Furthermore, this article highlights the potential for engineering exosomes to be used as a tool for cancer immunotherapy.

## 2 Exosome contents and immunotherapy

Exosomes carry diverse biomolecules, including lipids, nucleic acids (DNA and RNA), and proteins. Much like the variety of biomolecules found within cells, the cargoes contained within exosomes are also highly heterogeneous in type and quantity, referred to as cargo heterogeneity of exosomes. When exposed to various stresses, the composition of exosomal cargoes can change, leading to alterations in the biological functions acquired by recipient cells. For example, hypoxia can upregulate 130 and downregulate 129 proteins in exosomes from non-small cell lung cancer A549 cells, demonstrating that stress can enhance the diversity of exosomal cargoes (Mo et al., 2020). Exosomal ANGPLT4 is positively related to A549 cell migration in different oxygen levels. However, even when treated with exosomes from hypoxic ANGPLT4-KD cells, the migration abilities of A549 cells were still higher than those treated with exosomes under normoxia. These findings suggest that exosomal cargoes may function in a complementary manner to facilitate their biological functions (Mo et al., 2020). Due to the heterogeneity of exosomal cargoes, exosomes carrying specific cargoes can mediate various immune reactions. In this article, we will focus on the roles of three main exosome cargoes: proteins, lipids, and nucleic acids, in the context of tumor immunity (Table 1).

**TABLE 1:** Exosome contents and immunotherapy.

Type of molecule	Function in tumor immune microenvironment	Exosome source cells	Exosome receptor cells	Ref.
Proteins	Exosomal HMGB1 from hepatocellular carcinoma cells promotes TIM-1^+^ regulatory B cell expansion via HMGB1-TLR2/4-MAPK pathways, which produce immunosuppressive cytokine IL-10 and can suppress CD8^+^ T cell activity	Hepatocellular carcinoma cells	TIM-1^+^ regulatory B cells	Ye et al. (2018)
	CSF-1 bearing EVs from triple negative breast cancer cells induce monocyte differentiation toward proinflammatory macrophages with IFN response partly through the cGAS/STING pathway and are associated with better clinical outcomes	Triple-negative breast cancer cells	Monocytes	Tkach et al. (2022)
	Activated T cell-derived exosomal PD-1 can enhance antitumor immunity in triple-negative breast cancer	Activated T cells	PD-L1 expressing metastatic TNBC cells	Qiu et al. (2021)
	Exosomal TNF-α from NK cells exert cytotoxic effects on melanoma cells	NK cells	Melanoma cells	Zhu et al. (2017)
	Exosomal FasL from T cells can promote invasion and metastasis of Fas^+^ tumor cells via increasing the expression of MMP9	T cells	Fas^+^ tumor cells	Cai et al. (2012)
	CD73-expressing exosomes from Treg cells modulate T cells into a suppressive state	Treg cells	T cells	Smyth et al. (2013)
RNA	TAM-derived exosomes containing miR-21-5p and miR-29-3p induce Treg/T helper (Th)17 cell imbalance by suppressing STAT3, thus facilitating the metastasis of epithelial ovarian cancer cells	TAMs	Treg cells and Th17 cells	Zhou et al. (2018)
	Exosomal miR-365 from TAM increases the resistance of PDAC cells to gemcitabine	TAMs	PDAC cells	Binenbaum et al. (2018)
	LncRNA TUC339 from hepatocellular carcinoma cells-derived exosomes induces the polarization of target macrophages from M1 (antitumor cells) to M2 (pro-tumor cells)	Hepatocellular carcinoma cells	Macrophages	Li et al. (2018)
	Breast cancer cell-derived exosomes carrying miR-130 target macrophages and polarize them to the M1 phenotype, inducing proinflammatory cytokine production and inhibiting cancer	Breast cancer cells	Macrophages	Moradi-Chaleshtori et al. (2021)
Lipids	Ceramide can trigger exosome biogenesis as an ESCRT-independent production pathway, and GW4869 (the neutral sphingomyelinase inhibitor) has been widely used to reduce the release of exosomes via inhibiting ceramides	-	-	Trajkovic et al. (2008)
	The acidic and hypoxic tumor microenvironment reshapes the composition of exosomal lipids to change the uptake of exosomes by tumor cells	Tumor cells	-	Gong et al. (2021)
	Tumor-derived exosomal lipids directly result in the dysfunction of dendritic cells to accomplish immune evasion	Tumor cells	Dendritic cells	Yin et al. (2020)

### 2.1 Proteins

Proteins are the functional components of exosomes, playing a pivotal role in mediating their biological functions. Although the mechanisms underlying the loading of proteins into exosomes remain unclear, numerous studies have demonstrated that proteins carried by exosomes play essential roles in regulating the functions of immune cells involved in tumor immunity (Zhang and Yu, 2019; Morrissey and Yan, 2020; Wang G. et al., 2021; Pathania et al., 2021). Exosomes derived from tumor cells have been shown to impact immune cells involved in tumor immunity. On the one hand, exosomal proteins derived from tumor cells can promote a pro-tumor immune status (Greening et al., 2015; Gao et al., 2018; Li et al., 2020). For instance, exosomal HMGB1 derived from hepatocellular carcinoma cells has been shown to encourage the expansion of TIM-1+ regulatory B cells via the HMGB1-TLR2/4-MAPK signaling pathway. These regulatory B cells produce immunosuppressive cytokines, such as IL-10, which can suppress the activity of CD8^+^ T cells, ultimately promoting tumor progression (Ye et al., 2018). Moreover, the secretion of HMGB1 can be inhibited by a specific exosome inhibitor called GW4869 (Kim et al., 2021). However, exosomes are essentially bio information messengers and a subtype of EVs, and their particular functions depend on the cargoes they carry (Gehrmann et al., 2014; Koh et al., 2017; Wang C. et al., 2021; Yin et al., 2021). On the other hand, exosomes derived from tumor cells may also inhibit tumor development through the immune system. For instance, exosomes bearing CSF-1 from triple-negative breast cancer cells can induce monocyte differentiation towards proinflammatory macrophages with IFN response, partly through the cGAS/STING signaling pathway, and are associated with better clinical outcomes (Tkach et al., 2022). Secondly, exosomal proteins from immune cells can also regulate tumor progression by influencing cells within the tumor microenvironment, including immune and tumor cells. For instance, exosomal PD-1 derived from activated T cells has been shown to attenuate PD-L1-induced immune dysfunction and enhance antitumor immunity in triple-negative breast cancer (Qiu et al., 2021). Exosomal TNF-α derived from natural killer (NK) cells has also been shown to exert cytotoxic effects on melanoma cells by blocking the cell proliferation signaling pathway (Zhu et al., 2017). These findings demonstrate that exosomal proteins can mediate the tumor-killing properties of immune cells. However, not all exosomal proteins derived from immune cells inhibit tumor progression; some may even facilitate tumor development. For instance, exosomal FasL derived from T cells has been shown to promote invasion and metastasis of Fas + tumor cells by increasing the expression of MMP9 (Cai et al., 2012). Indeed, Treg cells are known to release CD73-expressing exosomes that can modulate T cells into a suppressive state, thereby promoting tumor development (Smyth et al., 2013). The results above demonstrate that exosome proteins are essential mediators of communication between immune and tumor cells. Depending on their specific cargoes, the diverse range of proteins contained within exosomes can either promote or inhibit tumor development.

### 2.2 RNA

RNA carried by exosomes includes both messenger RNA (mRNA) and non-coding RNA (ncRNA), which encompasses a variety of RNA subtypes such as long non-coding RNAs (lncRNAs), small nuclear RNAs (snRNAs), microRNAs (miRNAs), vault RNAs, repetitive element RNAs, and so on (Valadi et al., 2007; Pegtel and Gould, 2019). Like proteins, exosomal RNA also plays a vital role in the tumor immune microenvironment. Exosomal RNA triggers biological functions similar to proteins, activating and inhibiting immune cells in the tumor microenvironment. Furthermore, the donor and target cells of exosomal RNA within the tumor microenvironment include both tumor and immune cells (He et al., 2022). For example, tumor-associated macrophages secreting miR-21-5p and miR-29-3p-positive exosomes can regulate Treg/T helper (Th)17 cell imbalance and promote tumor metastasis by inhibiting STAT3 Epithelial ovarian cancer cells (Zhou et al., 2018). Moreover, exosomal miR-365 derived from TAM has increased the resistance of pancreatic ductal adenocarcinoma (PDAC) cells to gemcitabine chemotherapy (Binenbaum et al., 2018). Additionally, lncRNA TUC339 derived from hepatocellular carcinoma cell-derived exosomes have been shown to induce the polarization of target macrophages from M1 (antitumor cells) to M2 (pro-tumor cells) (Li et al., 2018). On the other hand, breast cancer cell-derived exosomes carrying miR-130 have been shown to target macrophages and polarize them towards the M1 phenotype, inducing proinflammatory cytokine production and inhibiting cancer (Moradi-Chaleshtori et al., 2021). Indeed, more and more research is being conducted on exosomal RNAs, and harnessing their potential for tumor treatment appears promising. For instance, loading exosomes with antisense oligonucleotides targeting STAT6 and using these exosomes in combination with immune therapy has shown great antitumor effectiveness in mice, resulting in over 90% tumor growth inhibition and 50%–80% complete remissions (Kamerkar et al., 2022). Given the promising results observed in preclinical studies, it is likely that exosomal RNAs will enter clinical trials and eventually improve tumor treatment. The ability of exosomes to efficiently deliver specific cargo, including RNAs, to target cells within the tumor microenvironment makes them an attractive tool for cancer therapy. However, further research is needed to fully understand the mechanisms underlying exosomal RNAs’ actions and optimize their therapeutic potential in the clinic.

### 2.3 Lipids

In essence, exosomes are organelles surrounded by a single lipid bilayer membrane and typically contain a variety of lipids. These lipids help maintain the exosome membrane’s structure and integrity and play essential roles in facilitating intercellular communication between exosome donor and recipient cells (Pegtel and Gould, 2019). Research on exosomal lipids has been relatively limited compared to proteins and RNAs. However, exosomal lipids are essential in the biology of exosomes. Lipids present on the membrane of exosomes can influence the biogenesis of exosomes. For instance, ceramide has been shown to trigger the biogenesis of exosomes by activating an ESCRT-independent pathway, leading to the formation of ceramide-rich multivesicular bodies that ultimately generate exosomes (Trajkovic et al., 2008). GW4869 has been widely used as a neutral sphingomyelinase (nSMase) inhibitor to reduce the release of exosomes by inhibiting ceramide synthesis. By inhibiting nSMase, GW4869 reduces the levels of ceramides and thus interferes with ceramide-dependent exosome biogenesis, ultimately leading to reduced exosome release. Therefore, lipids on exosome membranes play essential roles in regulating the biogenesis and release of exosomes (Trajkovic et al., 2008). In addition to their role in biogenesis, exosomal lipids can influence the uptake of exosomes by recipient cells within the tumor microenvironment. Gong et al. found that the acidic and hypoxic tumor microenvironment can reshape the composition of exosomal lipids, ultimately affecting the uptake of exosomes by tumor cells. These changes in lipid composition may alter the biophysical properties of exosomes, making them more or less likely to be taken up by target cells (Gong et al., 2021). Furthermore, tumor-derived exosomal lipids have also been shown to directly contribute to immune evasion by inducing the dysfunction of dendritic cells. For example, tumor-derived exosomes carrying high levels of ceramides have been shown to impact dendritic cell function negatively, impairing antigen presentation and reducing activation of T cells, ultimately leading to an immunosuppressive tumor microenvironment that aids tumor growth and progression (Yin et al., 2020). Indeed, great interest is in targeting exosomes and engineering them to carry specific cargoes to modulate tumor immune responses. These advances can potentially revolutionize cancer treatment by delivering therapeutic payloads directly to target cells within the tumor microenvironment while avoiding toxicities associated with traditional systemic therapies. With ongoing research efforts, we can expect to see significant progress in this field and the development of novel exosome-based therapeutics for cancer patients shortly.

## 3 Exosome membrane molecules and immunotherapy

### 3.1 The role of exosome PD-L1 in tumor immunotherapy

The outer layer of exosomes is composed of a lipid membrane that not only contains biological molecules such as proteins, DNA, and RNA but also expresses specific proteins on the surface of the membrane. One of the most common proteins expressed on the covers of exosomes is PD-L1 (Chen et al., 2018; Hu et al., 2022). PD-L1 is a ligand of PD-1, which negatively regulates the antitumor immune (Yi et al., 2021). However, when PD-L1 expressed on the surface of tumor cells binds with PD-1 on immune cells such as T cells, inhibitory signals are transmitted, ultimately resulting in a downregulation of the immune response and allowing tumor cells to evade immune detection and destruction. This immune suppression eventually leads to uncontrolled proliferation and growth of tumor cells within the body (Sun et al., 2018). Researchers use PD-1/PD-L1 immune checkpoint inhibitors, such as avelumab, atezolizumab, durvalumab, nivolumab, and pembrolizumab (Shen and Zhao, 2018; Korman et al., 2022). These drugs can bind to either PD-1 or PD-L1, blocking the inhibitory effect of tumor cells on immune function and restoring the activity of T cells, ultimately leading to a more robust immune response against the tumor and improved outcomes for patients with cancer (Shen and Zhao, 2018; Korman et al., 2022).

#### 3.1.1 Exosomal PD-L1 induces resistance to immune checkpoint therapy

**FIGURE 1:**
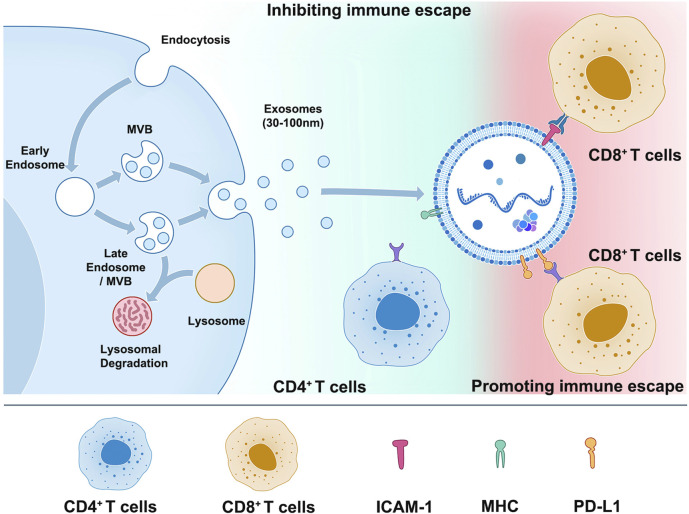
Exosome membrane molecules and immunotherapy.

While PD-1/PD-L1 immune checkpoint inhibitors have improved the prognosis of many cancer patients, most patients do not respond to this therapy or experience a loss of response over time. Understanding the underlying mechanisms behind this lack of response is an active research area. Recent evidence suggests that the expression of PD-L1 on the surface of exosomes may be one reason for the low efficacy of anti-PD-L1/PD-1 therapy, including glioblastoma, nonsmall-cell lung cancer, head and neck squamous cell carcinoma, prostate cancer, oral-oesophageal cancer, and colorectal cancer (Yin et al., 2021). Exosomal PD-L1 can contribute to systemic immunosuppression by engaging with PD-1 receptors on immune cells, inhibiting T cell activation and function. Additionally, exosomal PD-L1 may allow tumor cells to evade immune surveillance even in the presence of PD-1/PD-L1 checkpoint inhibitors, ultimately leading to treatment resistance (Table 2) (Yin et al., 2021). Indeed, Mauro Poggio et al. recently explored the role of exosomal PD-L1 in tumor progression by knocking down two essential genes related to exosome formation, Rab27a, and nSMase2, as well as PD-L1 itself. Their study revealed the critical role of exosomal PD-L1 in tumor cell immune evasion, suggesting that targeting exosomal PD-L1 may be a promising strategy for improving the clinical benefit of PD-1/PD-L1 checkpoint inhibitor therapy. This research highlights the importance of studying all aspects of exosomes, including their lipid and protein components, to develop more effective cancer therapies (Poggio et al., 2019). The data obtained from prostate cancer and colorectal cancer models suggest that inhibiting the release of PD-L1 in exosomes could be a promising treatment strategy for overcoming resistance to immune checkpoint inhibitors (Poggio et al., 2019). Whether used alone or combined with current immune checkpoint inhibitors, this approach can improve the response rate of patients who do not independently respond to checkpoint inhibitor therapy. These findings provide a strong rationale for further research into targeting exosomal PD-L1 as a novel therapeutic strategy in cancer treatment (Poggio et al., 2019). Similarly, in a breast cancer model, Yang et al. used Rab27A knockdown and the exosome inhibitor GW4869 in combination with anti-PD-L1 drugs. This combination therapy significantly enhanced the efficacy of immune checkpoint therapy, suppressed tumor growth, and restored tumor immunity. These results further support the potential of targeting exosomes as a novel therapeutic strategy for enhancing the effectiveness of current checkpoint inhibitor therapy and improving outcomes for patients with cancer (Yang et al., 2018). The mechanisms underlying drug resistance to exosomal PD-L1 are not yet fully understood, but two main hypotheses have been proposed: 1. Exosomal PD-L1 can bind to anti-PD-L1 antibodies, resulting in a reduction of PD-L1 expression on the cell membrane surface that is targeted by the antibody, which may contribute to the development of drug resistance and reduced efficacy of anti-PD-L1 therapy. 2. Although anti-PD-L1 antibodies can bind to PD-L1 on the cell membrane surface, exosomal PD-L1 can still bind to PD-1 receptors on T cells and inhibit their function, which suggests that targeting exosomal PD-L1 may be necessary to overcome drug resistance and enhance the effectiveness of immune checkpoint inhibitor therapy. Further research is needed to fully understand these mechanisms and develop effective strategies for targeting exosomal PD-L1 in cancer treatment (Yin et al., 2021; Hu et al., 2022) (Figure 1).

**TABLE 2:** Exosome membrane molecules and immunotherapy.

Exosome membrane molecule	Role in tumor immunotherapy	Ref
PD-L1	Negatively regulates immune cells, can develop resistance to immune checkpoint therapy, and can serve as a marker of immunotherapy response	Yang et al. (2018); Poggio et al. (2019); Yin et al. (2021); Hu et al. (2022)
MHC	Can retain MHC molecules on DC cell membrane to promote tumor immunity	Matsumoto et al. (2004); Escudier et al. (2005); Segura et al. (2005)
ICAM-1	Mediates adhesion between tumor exosomes and CD8^+^ T cells; blocking expression can increase infiltration and activation of CD8^+^ T cells in tumors and improve the efficacy of immunotherapy	Skokos et al. (2001); Segura et al. (2005); Zhang et al. (2022)

Indeed, Gong et al. recently identified two unique secretory PD-L1 shear variants lacking transmembrane regions in non-small cell lung cancer (NSCLC) patients resistant to PD-L1 antibody treatment. These variants serve as a new resistance mechanism to PD-L1 antibody treatment and can act as “bait” for PD-L1 antibodies, reducing their effectiveness in immune checkpoint therapy. In a co-culture system of HLA-matched iPSC-derived CD8^+^ T cells and cancer cells, the secreted PD-L1 variants were shown to inhibit the activation and proliferation of T cells, ultimately leading to reduced efficacy of immune checkpoint inhibitor therapy. These findings highlight the importance of studying the complex interactions between tumor cells and the immune system and developing novel strategies to overcome drug resistance in cancer treatment (Gong et al., 2019).

No drug is available to inhibit exosomal PD-L1 in the clinic specifically. However, targeting exosomes and their cargo, including exosomal PD-L1, represents a promising strategy for improving the efficacy of immune checkpoint inhibitor therapy and overcoming drug resistance in aggressive tumors currently unresponsive to immunotherapy. The development of new therapeutic methods based on these approaches has the potential to improve outcomes for patients with cancer significantly. It represents an exciting area of research in cancer immunotherapy.

#### 3.1.2 Exosomal PD-L1 as a marker of tumor immunotherapy

Advancements in multiple immunohistochemistry technologies, high-throughput sequencing, and microarray technology have led to a deeper understanding of the tumor immune microenvironment (TIME) and the development of biomarker models for predicting the efficacy of immune checkpoint inhibitor therapy based on tumor and micro environmental factors. These models can consider various factors, including levels of immune cell infiltration, expression of immune-related genes, and specific mutations or alterations in tumors and surrounding tissues. By combining these different factors, researchers have developed more accurate predictors of response to immune checkpoint inhibitors, ultimately leading to better outcomes for patients with cancer. As these technologies continue to advance, we can expect further improvements in our ability to predict and treat cancer based on its unique TIME characteristics (Zeng et al., 2021; Chowell et al., 2022). Recent studies have identified several biomarkers that can predict the efficacy of immunotherapy, including familiar PD-L1 overexpression, microsatellite instability (MSI), and tumor mutation burden (TMB) (Chan et al., 2019; Doroshow et al., 2021; Kwon et al., 2021).

Furthermore, Chen et al. found that the pre-treatment exosomal PD-L1 level in melanoma patients who responded to anti-PD-1 drug (pembrolizumab) treatment was significantly lower than in non-responsive patients (Chen et al., 2018). This study provides strong evidence suggesting that exosomal PD-L1 may be a biomarker for predicting response to immunotherapy. By measuring exosomal PD-L1 levels before treatment, clinicians can identify which patients are most likely to respond to immune checkpoint inhibitor therapy and adjust treatment strategies accordingly (Chen et al., 2018). These findings are significant in developing personalized cancer treatment strategies based on individual patient characteristics and tumor biology. Similarly, Del Re et al. found that the level of PD-L1 in plasma-derived exosomes was significantly decreased in patients who responded to anti-PD-1 treatment (Del Re et al., 2018). This study also demonstrated the clinical feasibility of dynamically measuring PD-L1 expression in plasma-derived exosomes as a potential biomarker for predicting response to immune checkpoint inhibitor therapy. By monitoring changes in exosomal PD-L1 levels throughout treatment, clinicians can expect treatment response early on and adjust treatment strategies accordingly (Del Re et al., 2018). These findings highlight the potential utility of exosomal PD-L1 as a biomarker for predicting response to immunotherapy and monitoring disease progression in cancer patients.

Although anti-PD-1 therapy has been approved for cancer treatment, the prediction of immune response solely based on a single indicator is often challenging due to the complex and multifaceted nature of immune reactions. Consequently, the reliability of PD-L1 expression in tumor tissue as a stand-alone predictor of treatment response is compromised. Further research focusing on comprehensive profiling and integration of multiple factors will be crucial for developing more accurate predictive models and personalized treatment strategies in the field of cancer immunotherapy. Therefore, Zhang et al. predicted the efficacy of anti-PD-1 treatment by combining exosomal PD-L1 and CD28 levels. Before treatment, low-level exosomal PD-L1 and high-level CD28 were markers of high response to anti-PD-1 (Zhang et al., 2020).

Quantitative detection methods for PD-L1 derived exosomes have been developed to facilitate their clinical application in tumor immunotherapy. One such method is the Holmes-exopod-l1, a novel approach for detecting exosomal PD-L1 protein. This method utilizes an MJ5C aptamer designed specifically to bind to PD-L1 protein on exosome membranes, unaffected by the glycosylation of PD-L1 protein. In this technique, fluorescent-labeled MJ5C aptamer and exosomes are co-incubated in capillary tubes, and an infrared laser creates a micron-level temperature gradient. The thermophoresis rates of the free MJ5C aptamer and the exosomal MJ5C aptamer complex differ significantly, resulting in the faster depletion of the free aptamer from the reaction system. Consequently, the fluorescence intensity of the labeled aptamer can be measured under excitation light to determine the PD-L1 protein content of exosomes (Huang et al., 2020). Another method, surface-enhanced Raman scattering (SERS), has also been employed to detect exosomal PD-L1 protein. SERS is a susceptible fingerprint spectroscopy technology widely used in the biomedical field (Vendrell et al., 2013). Pang et al. utilized TiO2-modified magnetic beads to nonspecifically adsorb exosomes in plasma, followed by modifying nanoparticles with SERS probes connected to PD-L1 antibodies. Subsequently, exosome samples were incubated with gold nanoparticles to form a magnetic bead-exosome-gold nanoparticle complex structure. This complex was then enriched and separated using a magnetic field and analyzed using a laser confocal Raman spectrometer to detect the content of PD-L1 positive exosomes (Pang et al., 2020). The researchers also developed a multifunctional peptide as a probe for PD-L1 exosome sensing experiments and demonstrated that the SPR sensor had a detection limit of 16.7 particles/ml for PD-L1 exosomes. The reliability and practicality of this sensor were further confirmed through the analysis of exosomal PD-L1 levels in human serum samples (Wang et al., 2022). These innovative detection methods allow for the quantification of PD-L1 derived exosomes, enabling researchers and clinicians to accurately assess their presence and abundance. This information is vital for evaluating the effectiveness of tumor immunotherapy and developing personalized treatment strategies. Although these techniques are still being refined and validated, they hold promise in enhancing our understanding of exosomal PD-L1 and its role in cancer therapy. Further research and clinical validation are necessary to establish their clinical utility and broader application.

### 3.2 The role of exosomal MHC in tumor immunotherapy

MHC (major histocompatibility complex) molecules are a class of cell surface proteins that are widely present in vertebrates (Axelrod et al., 2019; Cho et al., 2021). They play a crucial role in the immune system by aiding in the recognition and presentation of antigens to T cells. MHC molecules are divided into two classes: MHC-I and MHC-II (Axelrod et al., 2019; Cho et al., 2021). MHC-I molecules are primarily expressed on almost all nucleated cells, while MHC-II molecules are predominantly expressed on antigen-presenting cells such as macrophages, B cells, and dendritic cells. The main function of MHC molecules is to activate T cell immune responses through the process of antigen presentation (Axelrod et al., 2019; Cho et al., 2021). When a cell undergoes infection or mutation, MHC-I molecules bind to abnormal peptide segments and present them on the cell surface, thereby triggering a response from CD8^+^ T cells (Cho et al., 2021). On the other hand, MHC-II molecules capture exogenous antigens, bind them to MHC-II molecules, and present them on the surface of antigen-presenting cells to activate CD4^+^ T cell responses (Axelrod et al., 2019).

Dendritic cells (DC) are the most functional professional antigen-presenting cells. After binding the MHC on its surface and the T cell receptor (TCR) on the surface of T cells, DC can efficiently and maintaining the immune response (Wculek et al., 2020; Jhunjhunwala et al., 2021). As a membrane protein, MHC can also be highly expressed in its secreted exosomes (Table 2). First, it has been found that DC cell-derived exosomes can bind to TCR through MHC-Ⅱ to induce NF-κB activation, promoting the survival of CD4^+^ T cells (Matsumoto et al., 2004). Segura et al. found through proteomic and biochemical analysis that mature DC exosomes enrich MHC II and intercellular adhesion molecule 1 (ICAM-1) to start naive CD4^+^ T cells (Segura et al., 2005).

Moreover, under such characteristics, DC exosomes have been used as a tumor vaccine for melanoma in phase I experiments, and their safety and effectiveness have been verified (Escudier et al., 2005). In addition, a phase II clinical trial initially showed that the clinical effect of IFN-γ-DEX loaded with MHC I and II positive exosomes after chemotherapy in NSCLC patients who could not undergo surgery (Besse et al., 2016). In conclusion, DC cell-derived exosomes may become effective drugs to be developed and widely used because they can retain MHC molecules on the DC cell membrane to promote tumor immunity (Figure 1).

### 3.3 The role of exosome surface adhesion proteins in tumor immunotherapy

The adhesion protein LFA-1 (Lymphocyte Function-Associated Antigen 1), an important cell surface protein belonging to the integrin family, is primarily found on lymphocytes and other white blood cells in the immune system, playing a crucial role in immune responses (Shi and Shao, 2023). LFA-1 is composed of an *a* chain (CD11a) and a *ß* chain (CD18), forming the αLβ2 receptor complex. This complex serves as a vital link in cell adhesion and intercellular interactions. LFA-1 binds to its ligand ICAM-1 (Intercellular Adhesion Molecule 1), mediating adhesion and mutual recognition between leukocytes and other cells. LFA-1 is involved in various immune cell functions and processes (Reina and Espel, 2017). Firstly, it plays a significant role in the migration and localization of lymphocytes. By binding to ICAM-1 on endothelial cells, LFA-1 facilitates lymphocyte rolling, adhesion, and transmigration, enabling directed movement and infiltration of immune cells (Reina and Espel, 2017). Secondly, LFA-1 plays a crucial role in antigen presentation, which allows T cells to interact with antigen-presenting cells (such as dendritic cells) and enhances antigen recognition and activation of T cells (Reina and Espel, 2017). Additionally, LFA-1 is a key factor in immune cytotoxicity (Reina and Espel, 2017). In natural killer cells and cytotoxic T cells, LFA-1 binds to ICAM-1 on target cells, promoting adhesion and the formation of immunological synapses, thereby enhancing the effectiveness of cytotoxicity (Gerard et al., 2021).

LFA-1, also known as α_L_β_2_ or CD11a/CD18, is the primary ligand of ICAM-1, mainly expressed on the surface of APC cells. T cells interact with APC cells during the formation of immune synapses, and the binding of LFA-1 and ICAM-1 occurs in the center of synapses before the binding of TCR and MHC (Gerard et al., 2021). ICAM-1 is not only expressed on the cell surface but also found to be expressed in DC and mast cell-derived exosomes (Skokos et al., 2001; Segura et al., 2005) (Figure 1). Zhang et al. showed that ICAM-1 on the surface of tumor exosomes mediates the adhesion between tumor exosomes and CD8^+^ T cells, and this adhesion is a prerequisite for the immunosuppressive effect mediated by tumor exosomes PD-L1, suggesting that tumor exosomes, as a complete functional unit, have a synergistic effect on their surface molecules (Zhang et al., 2022). At the same time, the study also pointed out that other adhesion molecules may be affected by interferon-γ and may be involved in regulating the interaction between tumor exosomes and CD8^+^ T cells (Zhang et al., 2022). Therefore, blocking the expression of ICAM-1 in tumor exosomes can increase the infiltration and activation of CD8^+^ T cells in tumors and improve the efficacy of immunotherapy.

This schematic illustrates the process of cell releasing exosomes (left) and the mechanism and importance of PD-L1, MHC, and ICAM-1 on the surface of exosomes in tumor immune escape (right). Tumor cells release exosomes containing high levels of PD-L1, which can bind to PD-1 on the surface of T cells and suppress T cell function, allowing tumor cells to evade immune surveillance and metastasize. Exosomes secreted by DC cells are rich in proteins such as MHC II, B7.2, and ICAM-1, which can bind to TCR on the surface of CD4^+^ T cells through MHC-Ⅱ and initiate natural immune responses. On the other hand, exosomes released by tumor cells have adhesion proteins such as ICAM-1 on their surfaces, which can bind to LFA-1 on the surface of CD8^+^ T cells and promote the interaction between tumor cell-derived exosomes and CD8^+^ T cells, leading to immune escape.

## 4 Engineered exosomes

### 4.1 Construction of engineered exosomes

The use of exosomes as diagnostic and therapeutic tools is gradually coming to the forefront as studies reveal their stability *in vivo*, low immunogenicity, permeability, and editability *in vitro* and *in vivo*. Genetic engineering has become one of the essential tools for editing and modifying exosomes. These genetic engineering are done by constructing targeting sequences on the surface of exosomes and then mosaic the cargo on the surface of exosomes or loading it into exosomes and evading microcellular drinking action in circulation, thus improving the targeting and effectiveness of tumor therapy.

In previous studies on exosome engineering, it has been reported that cadherin proteins present on the surface of exosomes can bind to exosomal lipids through their C1C2 structural domains. This study elaborated on the technique of exosome display, where an engineered fusion protein with the C1C2 structural domain is present in exosomes (Delcayre et al., 2005). Another study had a similar idea: cadherin was modified by the Gaussia fluorophore enzyme and overexpressed in B16-BL6 cells (Takahashi et al., 2013). After isolating the exosomes from the supernatant and administering intravenous injections to mice, Takahashi et al. utilized bioluminescence imaging to track the exosomes and visualize their presence in the mice. Other studies have taken another approach by fusing the cytoplasmic structural domain of the TMEnv protein from the bovine leukemia virus to the extracellular structural domain of CD8, resulting in CD8 enrichment in exosomes (De Gassart et al., 2009). These exosomal membrane protein-based studies have paved the way for subsequent studies to validate exosome targeting. Lysosomal-associated membrane protein 2 (LAMP2b) is an exosomal membrane protein expressed on mouse exosomes and is widely used for exosomes. A polypeptide was inserted after its N-terminal signal peptide to achieve fusion expression of the polypeptide with LAMP2b and to be packaged into exosomes. For example, targeting chronic granulocytic leukemia (CML) cells using LAMP2b fused to an IL-3 fragment allows exosomes to inhibit CML cell growth *in vitro* and *in vivo* by loading Imatinib or siRNA targeting bcr-abl into engineered exosomes (Bellavia et al., 2017). In addition, immature dendritic cells expressing the αv integrin-specific targeting peptide IRGD (CRGDKGPDC) were fused to Lamp2b to produce exosomes modified with IRGD. Loaded adriamycin-functionalized exosomes can effectively accumulate in αv integrin-positive breast tumors and lead to significant tumor collapse (Tian et al., 2014). A separate study gave an alternative approach using LAMP2b to demonstrate hepatopeptide tLyp-1 (CGNKRTR) as a binding to neurofibrillary protein 1/2 (NRP1/2) highly expressed on tumor cell membranes specific ligand (Bai et al., 2020).

In addition to uncovering exosomal membrane proteins for tracking exosomes and improving exosome targeting, today’s research also explores other proteins or peptides that can be carried on the exosome surface, thereby optimizing the ability of engineered exosomes to deliver active substances. For example, a comprehensive study identified proteins and peptides that could be carried on the surface of exosomes, a survey that coupled GFP to candidate proteins and performed functional analysis (Dooley et al., 2021). After optimizing exosome work, proteomics led to the identification of the prostaglandin F2 receptor negative regulatory prostaglandin F2 receptor negative regulator (PTGFRN), a gene that effectively carries GFP on the surface of exosomes (Dooley et al., 2021). Furthermore, this study completed the optimization process for truncated PTGFRN, which will serve as a guide for exosome editing (Dooley et al., 2021). Interestingly, this study used PTGFRN as a scaffold to carry IL12, which is closely related to inflammatory cell activation, on the exosome membrane, and constructed the engineered exosome exoIL12. And intratumoral injections of exoIL12 in a mouse MC38 tumor model showed more potent antitumor activity and longer half-life than recombinant IL12 (Lewis et al., 2021). The complete tumor response rate to exoIL12 was 63% (much higher than recombinant IL12), suggesting that exosomes could introduce many protein-based immune-related therapies into clinical (Lewis et al., 2021).

In addition to genetic engineering to load membrane proteins onto extracellular somatic membranes, chemical engineering can also couple peptides to exosomal membranes, for example, curcumin and superparamagnetic iron oxide nanoparticles (Spion) were added to exosomes enriched in mouse gliomas, resulting in a significant reduction in tumor size and increase in survival (Jia et al., 2018). In a study using 1,2-Distearoyl-sn-glycero-3-phosphorythanolamine and polyethylene glycol to link AA ligands into macrophage-derived exosomes, and paclitaxel was later loaded into the exosomes by ultrasound. The modified exosomes targeted lung metastases in a Lewis in non-small cell lung cancer mouse model and improved survival (Kim et al., 2018). Another study changed the exosomes to target DC by mannose-linked polyethylene glycol. To enhance the immune response in the presence of dimethyl sulfoxide, monophosphatidyl lipid A (adjuvant) was loaded into exosomes and caused exosomes to specifically target DCs and increase the tumor necrosis factor-α and interleukin 6 (Choi et al., 2019).

The exosomes can act like electronic cars to transport their contents to target cells without risk of degradation or accidental interactions. The researchers who implemented the “truncated PTGFRN” process mentioned above also investigated the proteins that transport cargo in the exosome (Dooley et al., 2021). They found that brain abundant membrane attached signal protein 1 (BASP1) is associated with the inner leaflet of the membrane. By truncating BASP1, they optimized and identified an octaamino acid peptide that specifically loaded GFP into exosomes, comparable to the full-length BASP2 protein (Dooley et al., 2021). In addition, it was shown that ExoOVA, an exosome carrying ovalbumin, successfully induced interferon-gamma (Dooley et al., 2021). In addition to editing exosomes to have proteins, these are some ways to edit exosomes to make nucleic acids. For example, the RNA-binding protein Human Antigen R (HuR) was introduced into the exosomal membrane protein CD9 luminal surface and then loaded with exosome miRNAs (Li et al., 2019). One such device involves introducing L7Ae into exosomes by splicing it to the C-terminus of CD63 and inserting the C/D box region into the 3′-untranslated region of a reporter gene encoding a bioluminescent reporter protein. These engineered exosomes could target the brain and deliver their contained mRNA (Kojima et al., 2018).

Whether it is exosome surface carriage or exosome intratumoral loading, the existing research on engineered exosomes provides new options and ideas for treating tumors. It paves the way for the clinical translation of engineered exosome-targeted therapies (Table 3).

**TABLE 3:** Examples of engineered exosome construction.

Type of engineering	Description	Target cells	Cargo	Effectiveness	Ref
Surface Protein-Based	Lactadherin-based fusion protein with C1C2 structural domain	B16-BL6 cells	Gaussia fluorophore enzyme	Successfully tracked exosomes by bioluminescence imaging in mice	Delcayre et al. (2005); Takahashi et al. (2013)
Surface Protein-Based	TMEnv protein fused to the extracellular domain of CD8	-	CD8	Improved targeting of tumor therapy	De Gassart et al. (2009)
Surface Protein-Based	LAMP2b fused to IL-3 fragment	CML cells	Imatinib or siRNA targeting bcr-abl	Inhibition of CML cell growth *in vitro* and *in vivo*	Bellavia et al. (2017)
Surface Protein-Based	LAMP2b fused to αv integrin-specific targeting peptide IRGD	αv integrin-positive breast tumors	Adriamycin-functionalized exosomes	Effective tumor collapse	Tian et al. (2014)
Surface Protein-Based	CGNKRTR	NRP1/2 highly expressed on tumor cell membranes	Hepatopeptide	Specific binding to tumor cell membranes	Bai et al. (2020)
Surface Protein-Based	Proteins/peptides optimized for carrying cargo on the exosome surface	-	GFP, PTGFRN, IL12, curcumin, Spion, monophosphatidyl lipid A	Successful delivery of functional substances to target cells	Jia et al. (2018); Kim et al. (2018); Choi et al. (2019); Dooley et al. (2021); Lewis et al. (2021)
Intraluminal Cargo-Based	BASP1 is associated with the inner leaflet of the membrane	-	GFP	Efficient loading of GFP into exosomes	Dooley et al. (2021)
Intraluminal Cargo-Based	HuR introduced into the luminal surface of CD9 exosomal membrane protein	Target cells *in vivo* and *in vitro*	Specific miRNAs	Reduced expression of target proteins	Li et al. (2019)

### 4.2 Application to immunotherapy

**FIGURE 2:**
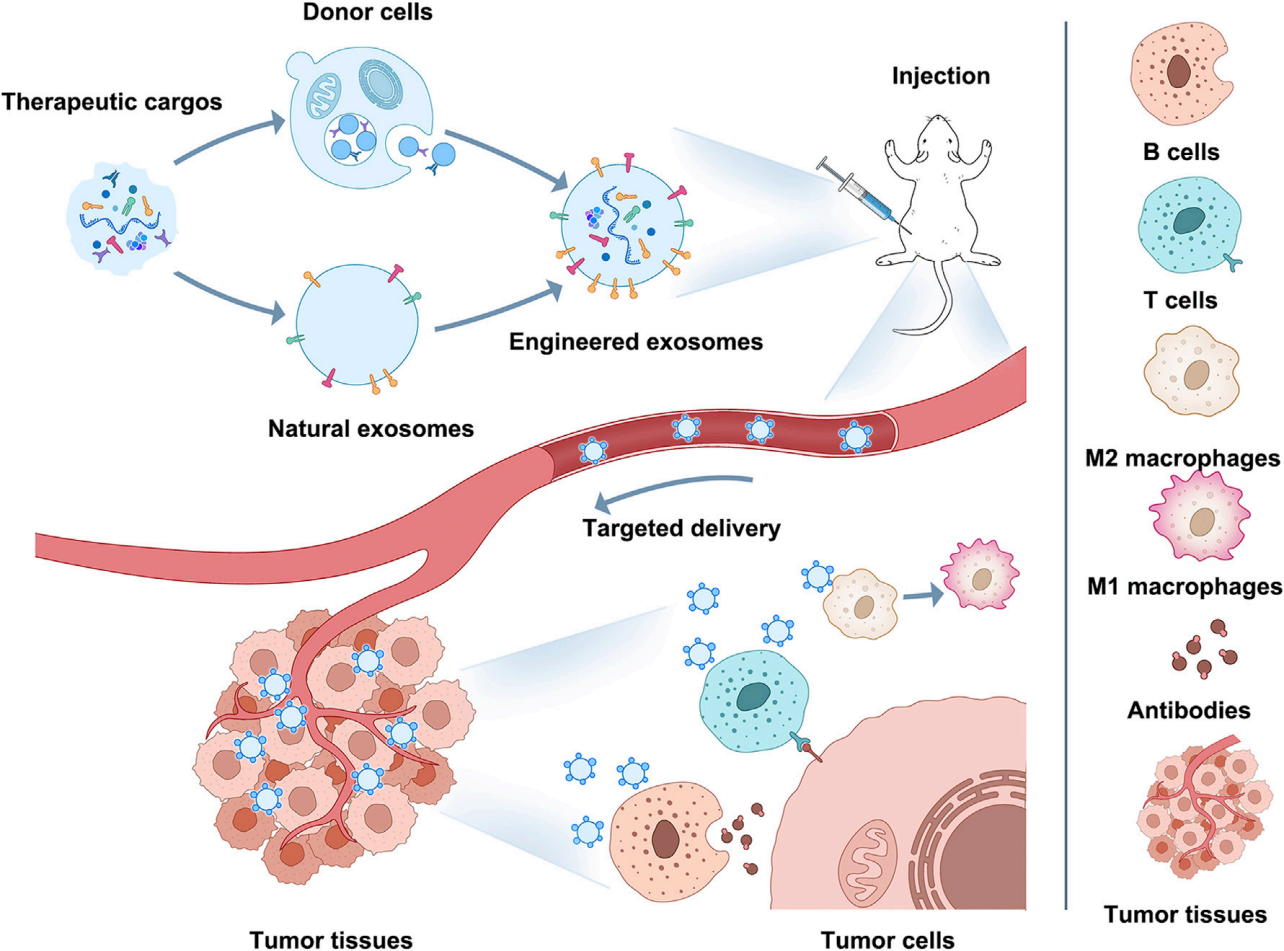
Application of engineered exosomes to immunotherapy.

Immune escape/immunosuppression is an essential contributor to the malignant progression of tumors, and changes in the phenotype and functional status of immune cells in the TIME affect both the speed of tumor progression and the outcome of tumors (Nagorsen and Thiel, 2006; Sabado et al., 2017; Galluzzi et al., 2020; Pulendran and Davis, 2020; Zhou H. et al., 2021). Therefore, immunotherapy based on immune cells and immune-related cytokines has become one of the mainstream approaches for tumor treatment (Srivastava et al., 2016; Suzuki et al., 2016; Lybaert et al., 2017; Truffi et al., 2019; Yang and Shah, 2020). And the current exosome modification technology involved in engineered exosomes could be a breakthrough point for improving immunotherapy through exosomes in the future (Figure 2).

Macrophages in the tumor microenvironment exhibit two distinct polarization states, M1 (classically activated) and M2 (alternatively activated) (Mantovani et al., 2004; Sica and Mantovani, 2012). In different malignancies, M2 macrophages are associated with treatment resistance, metastasis, and low survival (Germano et al., 2008; Sica et al., 2008; Shree et al., 2011; Qiu et al., 2018). Signal transducer and activator of transcription 6 (STAT6) is a crucial macrophage M2 transcriptional program regulator in various cancer conditions (Gordon and Martinez, 2010). TAM from Stat6−/− tumor-bearing mice exhibits an M1 phenotype and shows enhanced rejection of different tumor types (Kacha et al., 2000; Sinha et al., 2005). Therefore, this study constructed a novel engineered exosome exoASO-STAT6 that exhibited maximal biodistribution and STAT6 silencing activity in the liver with minimal effects on other tissues (Kamerkar et al., 2022). These exosomes significantly reduced the number of M2 macrophages and promoted an increased M1 antitumor phenotype accompanied by CD8^+^T cell-dependent activation of adaptive immune responses (Kamerkar et al., 2022). Researchers transformed M1-derived exosomes with miR-5a-3p and NK-kB siRNA transfection, attached IL4R-binding peptides to exosome membranes using DOPE-PEG amine, achieved targeted delivery of exosomes to M2-type macrophages, thereby driving M2 to M1 polarization and reducing tumor size in a 4T1 tumor model (Gunassekaran et al., 2021).

The antigen-presentation characteristics of dendritic cells are critical for inducing an immune response, and dendritic cell exosomes associated with antigen presentation have the potential to activate the immune system. In one study, dendritic cell-derived exosomes were modified by integrating respiratory syncytial virus antigens using MHC-I molecules on the surface of the exosomes, and *in vitro* success demonstrated their effective activation of CD8^+^ T cells (Hong et al., 2021). However, the exosomes failed to activate CD8^+^ T cells successfully, so further optimization is needed (Hong et al., 2021). K562 cells were genetically engineered as part of this process (Kim et al., 2017). In another attempt to activate the immune system using exosomes, researchers developed lactoadhesive and genetically engineered murine B16-BL6 melanoma cells with streptavidin fusion proteins (Morishita et al., 2016). After isolation of modified exosomes, they were incubated with an innate immune response activator (biotinylated CpG DNA) (Morishita et al., 2016). The results demonstrated that these engineered exosomes could activate DC2.4 cells and drive the antigen presentation process (Morishita et al., 2016).

Interestingly, a hybridization approach was used in one study to design exosomes for immunotherapy (Fan et al., 2022). This study initially collected dendritic cell exosomes induced by ovalbumin, expressing MHC necessary for T cells. These exosome membranes were then enriched with anti-CD3 and -EGFR receptors to enable the binding of the exosomes to T and B16-OVA cells. The exosomes could induce cellular toxicity by facilitating this connection between these cells and help both cells approach cytotoxicity (Fan et al., 2022). The described treatment using engineered exosomes resulted in an improved immune response when used in conjunction with PD-L1 inhibitors. PD-L1 inhibitors led to a reduction in tumor progression in a mouse B-16OVA model (Fan et al., 2022).

Editing exosome membrane surface proteins can facilitate engineered exosome transformation into immune reagents. A study demonstrated that using the C1C2 structural domain of cadherin to present tumor-associated antigens such as carcinoembryonic antigen (CEA) and human epidermal growth factor receptor 2 (HER2) on the surface of exosomes derived from antigen-presenting cells enhanced T and B cell responses, thereby promoting antitumor immune responses (Hartman et al., 2011). In another study, exosomes were converted into immune mediators through platelet-derived growth factor receptor (PDGFR). The PDGFR enabled the conversion of exosomes into immune mediators that could target both T cells and HER2-expressing cancer cells. The targeted T cells showed antitumor activity in a mouse breast cancer due to this dual targeting approach (Shi et al., 2020). In conclusion, the effectiveness and precision of engineered exosome-mediated tumor immunotherapy make exosome-based core pathway tumor immunotherapy occupy a place in future clinical oncology treatment.

Exosomes have made significant progress in clinical trials within the field of tumor immunotherapy. Several clinical trials are currently investigating their potential applications. For instance, NCT05559177 is a Phase I clinical trial targeting patients with recurrent or metastatic bladder cancer. The study aims to isolate dendritic cells or macrophages from peripheral blood and prepare personalized hybrid exosome vaccines for each patient. Additionally, other clinical trials are exploring the combined use of exosomes with other therapeutic approaches. One such example is NCT03985696, which focuses on diffuse large B-cell lymphoma patients resistant to rituximab plus cyclophosphamide, doxorubicin, vincristine, and prednisone (R-CHOP) therapy. This trial evaluates the safety and preliminary efficacy of exosomes in combination with immunotherapy. Another trial, NCT01159288, targets platinum-resistant lung cancer patients and investigates the role of tumor antigen-loaded dendritic cell-derived exosomes in improving chemotherapy resistance. These clinical trials aim to assess the safety, efficacy, and potential clinical applications of exosome-based treatments. Overall, ongoing clinical trials provide crucial insights into the potential applications of exosomes in tumor immunotherapy and lay the foundation for future development of innovative therapeutic strategies.

This Figure shows how engineered exosomes can be used for immunotherapy. Exosomes can be modified on their surface or intraluminally to carry specific cargo (proteins, peptides, nucleic acids) that can target tumor cells or modulate the immune response. For example, engineered exosomes can target M2 macrophages and induce M2-to-M1 polarization, activate CD8^+^ T cells and B cells, stimulate antigen-specific CD8^+^ T cells or kill tumors directly with b cell-derived antibodies, enhance tumor antigen presentation, and enhance antitumor immune responses. Using engineered exosomes as immune reagents can become a potential breakthrough for improving immunotherapy and occupying a place in future clinical oncology treatment.

## 5 Conclusion

This review explores exosomes as extramembrane active substances and their packaging contents to analyze their mode of regulation on the immune system. The core part focuses on the potential of exosomes as important messengers to carry unique cargo proteins, such as PD-L1, MHC, and ICAM-1, in the remodeling and treatment of the tumor immune microenvironment. The exploration of monoclonal antibodies against specific proteins or gene editing technology, followed by the concept of engineered exosomes, has been introduced as an important way to improve cancer immunotherapy. We elucidated the main mechanisms of engineered exosomes and subclassified exosomes according to material type. Editing exosomes can provide an effective means to enhance clinical immunotherapy strategies and may be a considerable driving force to promote exosomes in tumor diagnosis and treatment.

Although the use of exosomes as therapeutic tools holds promise, it is crucial to recognize their limitations. So far, the clinical efficacy of exosomes-based therapy in cancer patients has been relatively low. Despite encouraging preclinical studies, translating these findings into practical clinical outcomes remains challenging. Additionally, the isolation and purification of exosomes still present challenges. Current isolation techniques often result in the co-isolation of exosomes with other types of EVs, making it difficult to obtain high purity and homogeneity of exosome populations. This complicates the analysis of their characteristics and functions. Furthermore, a key issue lies in distinguishing free PD-L1 from PD-L1 bound to exosomes in biological fluids, considering the latter’s potential as a biomarker. The development of reliable detection methods or technologies to differentiate and quantify PD-L1 on exosomes is crucial for its effective utilization as a biomarker. Additionally, successfully achieving functional purification of exosomes, devoid of other exosomes and contaminants, poses a significant obstacle in transforming exosomes into viable therapeutic tools. Establishing standardized purification protocols and implementing strict quality control measures are essential to ensure their therapeutic effectiveness and safety. In summary, it is important to acknowledge the limitations associated with using exosomes as therapeutic tools. Overcoming challenges related to clinical translation, isolation and purification, and accurate detection and quantification of specific biomarkers will be pivotal in harnessing the full potential of exosomes for clinical applications. Enhancing clinical immunotherapy strategies through the editing of exosomes can be a significant driving force for the promotion of exosomes in tumor diagnosis and treatment.
